# Laser Speckle Flowgraphy (LSFG) in Age-Related Macular Degeneration and Diabetic Retinopathy: A Systematic Review of Recent Literature

**DOI:** 10.3390/jcm14248928

**Published:** 2025-12-17

**Authors:** Carlo Bellucci, Medea Virgili, Alessandra Romano, Salvatore Antonio Tedesco, Paolo Mora

**Affiliations:** Ophthalmology Unit, University Hospital of Parma, 43126 Parma, Italy

**Keywords:** laser speckle flowgraphy, retinal perfusion, choroidal blood flow, age-related macular degeneration, diabetic retinopathy, ocular hemodynamics

## Abstract

**Background**: Laser Speckle Flowgraphy (LSFG) is a non-invasive imaging technology that quantitatively evaluates retinal and choroidal blood flow by analyzing speckle patterns generated by laser light scattering. This systematic review summarizes the application of LSFG in two major degenerative retinal diseases: age-related macular degeneration (AMD) and diabetic retinopathy (DR). **Methods**: A comprehensive literature search (2010–2025) was conducted in PubMed, Cochrane Library and EMBASE according to PRISMA guidelines. Twenty-three studies including a total of 974 eyes (191 AMD, 783 DR) were analyzed. **Results**: In AMD, LSFG detected baseline reductions in choroidal and retinal perfusion in non-exudative disease, often extending beyond atrophic regions. Anti-VEGF injections produced acute reductions in MBR, particularly with brolucizumab, with partial recovery over time; drug-specific differences suggest a potential impact on geographic atrophy progression. In DR, LSFG revealed early microvascular dysfunction even in asymptomatic eyes. Retinal and choroidal MBR and blowout score correlated with HbA1c, DR severity, and inflammatory mediators. Intravitreal anti-VEGF therapy consistently reduced retinal and choroidal MBR and RFV, while conventional panretinal photocoagulation decreased choroidal flow and vascular caliber more robustly than patterned laser, reflecting oxygenation-driven VEGF modulation. Low baseline MBR predicted higher central macular thickness and reduced therapeutic response in diabetic macular edema. **Conclusions**: LSFG provides reproducible, rapid, and non-invasive quantitative insights into ocular hemodynamics across degenerative retinal diseases. Its integration into multimodal imaging may facilitate early diagnosis, support personalized management, and assist in the prognostic assessment of retinal and choroidal vascular disorders.

## 1. Introduction

Retinal blood flow is crucial to maintain the metabolic and functional integrity of the retina. Different retinal diseases, including diabetic retinopathy, retinal vein occlusion, neovascular complications related to age-related macular degeneration or other diseases, may alter retinal circulation, leading to potentially serious complications for vision [1,2,3]. Traditionally, retinal blood flow has been assessed using fluorescein angiography (FA) and indocyanine green angiography (ICGA), which remain the gold standard but are invasive, time-consuming, and rely on dye injection, resulting in one of the few ophthalmic diagnostic tests with contraindications [4]. To overcome these limitations, non-invasive imaging modalities have been developed: OCT angiography (OCT-A), which provides an instantaneous map of the retinochoroidal perfusion, (segmented by layers of interest), and Laser Speckle Flowgraphy (LSFG).

LSFG is a non-invasive technique providing real-time, quantitative assessment of ocular blood flow by analyzing speckle patterns generated by laser light scattering. It uses an 830-nm diode laser whose backscattered light, captured by a photodetector, produces a granular interference pattern (speckle pattern) analyzed across a rapid sequence of 118 consecutive frames (30 Hz, ~4 s acquisition) over a 21–22° field of view [5,6,7].

**Figure 1:**
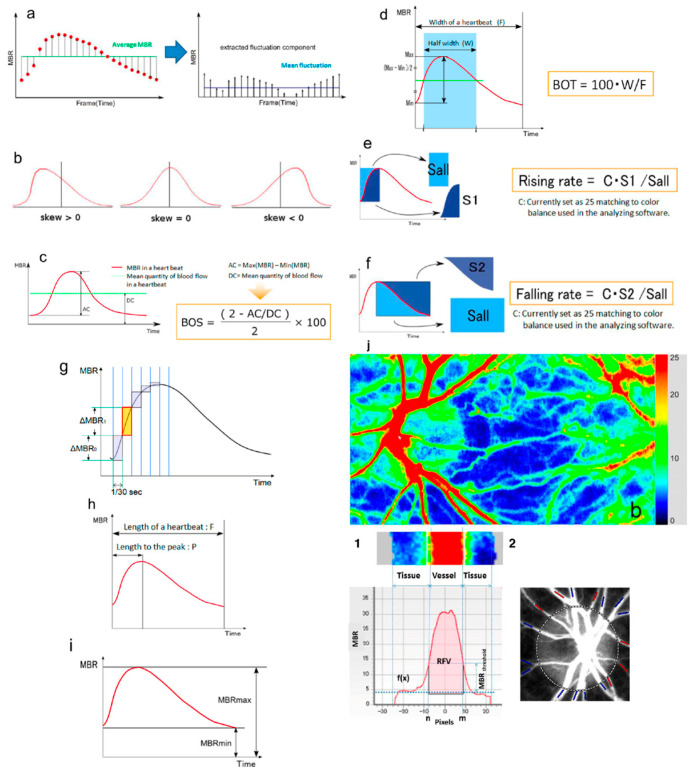
Schematic explanations of the parameters obtained from waveform analysis: (**a**) fluctuation, (**b**) skew, (**c**) blowout score (BOS), (**d**) blowout time (BOT), (**e**) rising rate, (**f**) falling rate, (**g**) flow acceleration index (FAI), (**h**) acceleration time index (ATI), and (**i**) resistivity index (RI). (**j**) Example of LSFG color map. (**1**) Determination of Retinal flow volume (RFV). (**2**) Blood vessels around the ONH are classified into “artery” (red) and “vein” (blue). Adapted with permission from Sugiyama, T. [5]. © 2014 by the author, licensed under CC BY 3.0 (https://creativecommons.org/licenses/by/3.0/. URL accessed on the 30 October 2025).

Blood flow is expressed as the mean blur rate (MBR) and relative flow volume (RFV). The motion of erythrocytes within retinal vessels causes speckle variations that are quantified as MBR, which can be further divided into MBR-vessel (MV, reflecting retinal circulation) and MBR-tissue (MT, mainly representing choroidal flow) [8,9,10]. The system averages all frames to generate pseudocolor flow maps visualizing ocular perfusion (Figure 1).

From the MBR waveform, additional hemodynamic parameters are derived: blowout time (BOT) and blowout score (BOS), describing flow stability during the cardiac cycle, and flow acceleration index (FAI) and acceleration time index (ATI), reflecting peak flow dynamics within one heartbeat. RFV estimates relative retinal blood flow by subtracting background choroidal flow from the total flow within a selected vascular region [11].

LSFG has become an increasingly valuable tool for the non-invasive evaluation of ocular blood flow. This systematic review summarizes recent literature on its role in age-related macular degeneration and diabetic retinopathy.

## 2. Materials and Methods

This systematic review was conducted in accordance with the PRISMA 2020 guidelines (see Supplementary Materials). Papers assessing ocular blood flow using LSFG in human subjects affected by age-related macular degeneration or diabetic retinopathy were included. Only peer-reviewed articles published in English between January 2010 and October 2025 were considered. Reviews, case reports, conference abstracts, and animal studies were excluded.

A comprehensive literature search was performed from July 2025 to October 2025 in PubMed, Cochrane Library and EMBASE. The search terms used were (“Laser Speckle Flowgraphy” OR “LSFG” OR “Laser Speckle Flow-Graphy”) AND (“age-related macular degeneration” OR “AMD” OR “diabetic retinopathy” OR “DR” OR “diabetic macular edema” OR “DME”)

The articles retrieved were independently reviewed by two authors (MV and CB), who examined all records by title and abstract, followed by a full-text assessment of the eligible studies. Disagreements were resolved by discussion with a third reviewer/author.

Data were extracted using a standardized form, including author, year, sample size, main outcomes and study design.

**Figure 2:**
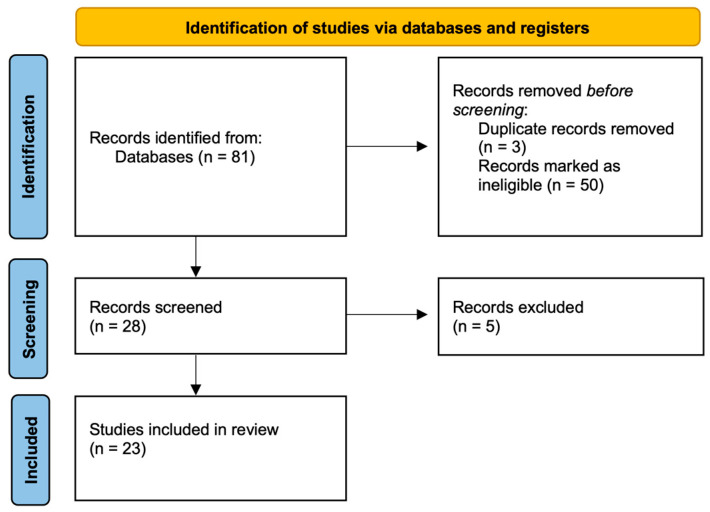
Systematic review flow diagram based on PRISMA guidelines.

The methodological quality of the included studies was assessed qualitatively, based on study design, sample size, clarity of outcome measures, and potential sources of bias (Figure 2). Case reports were excluded, while case series were included if they reported quantitative data relevant to the study outcomes. No formal risk-of-bias scoring system was applied, as the included studies were heterogeneous and primarily observational. This systematic review was not registered in PROSPERO or any other database.

Artificial intelligence (AI) tools were not used in the conduct of this systematic review and were limited to English language editing only.

## 3. Results

The search retrieved 81 papers, reduced to 23 after removal of duplications and ineligible works: 8 for age-related macular degeneration (AMD) and 15 for diabetic retinopathy (DR). The total number of eyes included in this review was 974: 191 for AMD, and 783 for DR.

### 3.1. LSFG and Age-Related Macular Degeneration

The earliest paper we reviewed was from Mursch-Edlmayr et al. (2019) [12]. This prospective study included 20 eyes affected by neovascular AMD (nAMD) treated with a single 2 mg Aflibercept intravitreal injection, comparing them to the fellow eye. Optic nerve head (ONH) perfusion was measured before and immediately after the intravitreal injection, after 30 and after 45 min. LSFG showed a decreased MBR, indicating a reduced perfusion of the ONH.

The same study group published a paper in 2020 [13], adopting the same inclusion criteria and treatment from their previous study. In this work, they employed LSFG to measure the long-term effect on retinal and choroidal circulation, measuring blood flow at baseline, 1 week after the first injection, at the time points of the second and third injection, and 1 month after the third injection. They found a prolonged reduction in perfusion of the ONH and the choroid of the treated eye, supporting the hypothesis that this could delay the development of geographic atrophy.

Calzetti et al. published two prospective studies in 2020 and 2021 [14,15]. In the first paper, they used LSFG to assess ocular perfusion within choroidal neovascularization (CNV) area in 13 eyes after intravitreal injection of Bevacizumab. They found a significant reduction in MBR after 1 week, which partially recovered after 1 month, suggesting a loss of the anti-VEGF effect over time. However, a progressive reduction in the central macular thickness (CMT) was observed.

In their second paper, the authors measured retinal and choroidal RFV to study the short-term effect of intravitreal injection of Bevacizumab in 10 eyes. They reported a significant reduction in both retinal and choroidal RFV within the first 10 days, followed by a partial recovery of the original choroidal blood flow within 1 month, while retinal RFV remained low.

Kato et al. (2023) [16] prospectively studied the short-term effects of intravitreal treatment with brolucizumab and aflibercept in 21 eyes of 21 patients affected by nAMD. The MBR of the ONH and choroid was measured before and 30 min after intravitreal injection. A statistically significant reduction in both choroidal and retinal blood flow was observed in both groups. No statistically significant difference was found between the two treatment groups; however, in 3 of the 10 eyes treated with brolucizumab, a decrease in blood flow greater than 30% was recorded.

In a retrospective study published in 2024 [17], Takizawa et al. measured ocular perfusion in 43 eyes with nAMD, of which 29 were treated with intravitreal brolucizumab and 14 with intravitreal ranibizumab. Blood flow was assessed before and one month after the injection, showing a significant reduction in MBR in both choroid and ONH in patients treated with brolucizumab, but not in those receiving ranibizumab. These findings suggest a short-term, drug-dependent effect on ocular perfusion. In both groups, a significant reduction in CMT was also observed.

Linton et al. (2025) [18] retrospectively compared 39 eyes from 24 patients with dry AMD to 41 eyes from 21 healthy subjects. They found a reduction in both choroidal (predominant) and retinal blood flow in patients with moderate to severe AMD. In some cases, this reduction extended beyond the areas of atrophy, supporting the role of impaired ocular perfusion in the development and potential progression of dry AMD.

In a recent retrospective observational study, Kato et al. (2025) [19] analyzed 45 eyes from 45 Japanese patients with nAMD treated with intravitreal injections of faricimab, brolucizumab, or aflibercept 2 mg. They evaluated choroidal and ONH MBR before and 30 min after the intravitreal injection. A significant reduction in MBR was observed in both the choroid and ONH across all three treatment groups, with no significant differences among them overall. However, eyes treated with brolucizumab showed a significantly greater decrease in choroidal blood flow compared to those receiving aflibercept.

### 3.2. LSFG and Diabetic Retinopathy

Nitta et al. (2014) [20] used LSFG technology to study 12 patients with diabetic macular edema (DME) and 13 patients with macular edema associated with branch retinal vein occlusion (BRVO). Among the 12 patients with DME, a reduction in MBR was observed in both retinal and choroidal blood flow, whereas in the 13 patients with BRVO, the MBR decreased only in the choroid. This finding may be due to the different pathophysiology of the edema. In DR, higher pre- and post-injection MBR at one month after a single intravitreal injection correlated with a thinner CMT. This suggests that intravitreal therapy may be less effective in cases with low baseline ONH MBR, indicating that MBR could serve as a biomarker for therapeutic decision-making in DME.

In their prospective study, Okamoto M. et al. (2015) [21] followed 24 eyes of 24 patients with severe non-proliferative diabetic retinopathy (NPDR) without macular edema for 12 weeks after panretinal photocoagulation (PRP). Both subfoveal choroidal thickness and choroidal MBR significantly decreased. Since PRP increases retinal oxygenation leading to reduced VEGF production, the observed thinning and reduced choroidal flow after PRP may indicate effective VEGF suppression and successful PRP treatment.

Shiba C. et al. (2016) [22] reported that LSFG can predict the functional status of the retinal and choroidal microcirculation and detect early changes even in asymptomatic cases. In this cross-sectional study, 196 patients were analyzed, 49 of whom had diabetes. Diabetic patients showed significantly lower choroidal MBR and BOS of both the ONH and choroid compared to non-diabetics. Among non-diabetics, HbA1c levels were negatively correlated with both choroidal and ONH BOS, as well as with choroidal MBR.

Iwase T. et al. (2017) [23] evaluated 76 eyes from 76 patients with severe NPDR who underwent PRP, 39 eyes of untreated NPDR patients, and 71 eyes of healthy controls. Patients who received PRP showed a significant reduction in ONH and choroidal MBR and a decrease in vascular lumen diameter.

In a prospective study from Sugimoto M. et al. (2017) [24], the effect of a single intravitreal injection of ranibizumab was measured in 15 patients with macular edema (11 secondary to DR and 4 secondary to BRVO). MBR was measured at baseline, 1 day, and 1 week after intravitreal injection. A significant reduction in MBR was observed in the treated eyes, with no significant changes in the fellow eyes.

Yamada et al. (2017) [25] investigated 35 eyes with proliferative and non-proliferative DR to assess the effect of PRP on ONH MBR and on the retinal flow velocity of the first retinal artery (RFV-A) and vein (RFV-V) before bifurcation, at baseline, and after 1, 3, and 6 months. Both RFV-A and RFV-V decreased significantly during follow-up, suggesting that LSFG can be a useful tool to evaluate PRP effectiveness.

Okamoto et al. (2018) [26] followed 28 eyes with DME treated with intravitreal ranibizumab and 20 healthy control eyes. The DME eyes were divided into those previously treated with PRP (*n* = 16) and those without PRP (*n* = 12). At baseline, eyes without PRP had a thicker subfoveal choroid. After the injection, choroidal blood flow decreased significantly only in the non-PRP group, where there was also a significant correlation between central retinal thickness and choroidal flow.

In their prospective study, Mikoshiba Y. et al. (2018) [27] included patients with severe NPDR to compare the effects of conventional PRP and patterned scanning laser (PASCAL) photocoagulation on choroidal circulation. Of the 39 eyes included, 17 eyes received PRP, 22 eyes received PASCAL, and 15 eyes were not treated. A significant reduction in choroidal MBR at the macular region was observed as early as 4 weeks after conventional PRP, while in the PASCAL group, this occurred only after 12 weeks. Subfoveal choroidal thickness significantly increased at 1 week and then decreased from week 8 onward, with no difference between groups.

In 2019 [28], the same research group (Iwase, T. et al.) reported that retinal vascular MBR decreased significantly after conventional PRP but not after PASCAL treatment.

Toto et al. (2020) [29] studied 40 eyes from 30 patients with non-proliferative DR, both treatment-naïve and treated with ranibizumab. They measured MBR and RFV at the ONH and peripapillary region at baseline, 2 weeks, and 1 month after IV injection. A significant reduction in perfusion and flow velocity with peripapillary vasoconstriction was recorded. Eyes with low baseline MBR values showed higher CMT.

Thirty-three treatment-naïve eyes with DME were analyzed by Mizui et al. (2020) [30], finding that both choroidal and retinal blood flow significantly decreased 1 month after ranibizumab injection. Retinal MBR showed a significant negative correlation with some inflammatory mediators, such as MCP-1 and IL-8, suggesting that retinal MBR may serve as a biomarker for treatment efficacy.

In their prospective study, Ueno T. et al. (2021) [31] examined vascular changes in 47 diabetic patients without retinopathy, 36 with moderate NPDR, 22 with severe NPDR, 32 with PDR, and 24 healthy controls. In DR, vascular walls were thicker and lumens narrower. In PDR patients, MBR was significantly lower than in healthy controls and other diabetic groups. However, no significant differences in MBR were observed among NDR, moderate NPDR, severe NPDR, and control eyes.

Saima Y. et al. (2024) [32] conducted the first LSFG-based study comparing aflibercept and faricimab in 15 eyes with DME previously treated with intravitreal aflibercept but switched to intravitreal faricimab after resistance was shown. Faricimab proved non-inferior to aflibercept in efficacy but offered a longer treatment interval (16 weeks). CMT improved after both aflibercept and faricimab, with no significant differences between drugs. ONH MBR significantly decreased after 1 week with both agents, but after 1 month, this reduction persisted only in the faricimab group.

Similarly, Mizukami et al. (2024) [33] compared the short-term effects of intravitreal aflibercept (20 eyes) and intravitreal faricimab (15 eyes) with DME for 1 month. From baseline to 1 month, CMT, retinal and choroidal MBR significantly decreased, with no significant differences between groups.

Abu El-Asrar et al. (2025) [34] analyzed the effect of PRP on 37 eyes of patients with proliferative and severe non-proliferative DR. After PRP, a significant reduction in retinal arteriole caliber and in ONH, choroidal, and tissue MBR was observed. At baseline, ONH and tissue MBR were significantly and negatively correlated with arteriovenous oxygen saturation difference. After PRP, a significant negative correlation emerged between choroidal MBR and arteriovenous oxygen saturation difference.

## 4. Discussion

LSFG is a non-invasive technology that allows quantitative in vivo measurement of blood flow in the microcirculation of the choroid, retina, and ONH. Its diagnostic role in degenerative retinal pathologies is becoming clear, providing the ability to quantify hemodynamic parameters that would otherwise be impossible to measure. Intravitreal anti-VEGF injections are widely used for treating ongoing CNV in AMD and, in theory, may induce changes not only in the neovascular lesion but also in choroidal blood flow. VEGF acts by activating nitric oxide synthase, producing a potent vasodilator important for choriocapillaris maintenance, as confirmed by in vitro studies [35]. Using LSFG, many authors confirm the effect of anti-VEGF drugs on reducing retinal and choroidal perfusion, which may be associated with an increased risk of developing geographic atrophy [12,13,14,15,16,17,18,19]. Discrepancies in choroidal blood flow results across studies appear due to differences in ROI location and flow measurement parameters. For example, Mursch-Edlmayr et al. and Kato et al. (2023) [12,16] differed in the ROI analyzed by LSFG, but both reported flow reductions with longer follow-up. Similarly, Calzetti et al. and Takizawa et al. [14,17] showed contrasting results, likely due to small patient numbers, different measurement parameters (MBR vs. RFV), and different anti-VEGF treatments (ranibizumab vs. bevacizumab). LSFG also allowed comparison of different agents, with brolucizumab showing a more pronounced decrease in blood flow compared to other drugs [16,17,19]. Lastly, widespread reductions in retinal and choroidal blood flow can be observed using LSFG in dry AMD, suggesting a pathogenic role of impaired perfusion [18].

LSFG and OCT-A provide complementary insights into AMD. LSFG quantifies perfusion, while OCT-A offers layer-resolved anatomical mapping, assessing vessel density in the choriocapillaris, superficial and deep capillary plexus, and visualizing CNV morphology. In dry AMD and geographic atrophy, OCT-A confirms early choriocapillaris dysfunction with vessel density loss extending beyond atrophic margins. In nAMD, anti-VEGF therapy reduces flow in the choriocapillaris and deep plexus, while the superficial plexus remains largely unaffected. Together, these modalities support the hypothesis that microvascular impairment is an early event in AMD and that anti-VEGF therapies induce layer-specific perfusion changes [10,36]. Further studies are needed to determine whether the extent and location of flow reduction can predict disease progression and whether enhancing perfusion could offer a therapeutic strategy. These studies will benefit from the integration of the information provided by OCT-A and LSFG.

OCT-A provides layer-specific anatomical mapping of the retinal microvasculature and excels in structural and topographic assessment. It enables visualization of microaneurysms, non-perfusion areas, capillary ischemia, and alterations in the superficial and deep capillary plexus. In contrast, LSFG provides a functional measure of blood flow, but can detect early pathological changes as well. HbA1c correlates negatively with choroidal and retinal BOS and choroidal MBR [21]. Flow changes in diabetics are debated: some studies report reduced choroidal MBR and retinal/choroidal BOS in diabetics [21], while others [22,28] find no difference in non-proliferative cases, with reductions occurring only in proliferative DR. Low MBR pre- and post-intravitreal injection correlates with poorer treatment response and higher CMT [26], and baseline retinal MBR negatively correlates with aqueous MCP-1 and IL-8, linked to vascular permeability and DME severity [27]. Many authors agree that intravitreal anti-VEGF therapy significantly reduces retinal and choroidal MBR and RFV [23,26,27,29,30]. Conventional PRP reduces choroidal thickness and retinal/choroidal flow [20,22,24], improving inner retinal oxygenation by destroying oxygen-demanding photoreceptors and lowering VEGF levels. Post-PRP MBR reductions indicate treatment efficacy [20]. PASCAL PRP reduces choroidal flow [24] but not retinal flow [25]. Comparing anti-VEGF treatments, aflibercept and faricimab show similar reductions in retinal/choroidal MBR, central retinal thickness, and improved visual acuity. Faricimab may have a prolonged effect, though longer follow-up is needed to confirm [29,30].

Applications of AI in OCT-A are already effective, encompassing image processing, automated quantification, abnormality detection, disease prediction, and longitudinal monitoring [37]. In contrast, integration of LSFG with AI, although very promising, is less advanced and may suffer from the lack of a population-based normative database for the parameters obtained by LSFG examination. Further improvements in device performance and parameter characterization are required to fully realize its integration with the AI potential.

This review has several limitations. The included studies are mainly small-scale, observational, and heterogeneous in methodology and patient selection. There was no standardized LSFG protocol across studies, which limits direct comparison of results. Furthermore, the absence of meta-analysis is due to the lack of homogeneous quantitative data. Despite these limitations, LSFG emerges as a valuable non-invasive tool for quantifying choroidal and retinal blood flow, offering insights into pathophysiology, early diagnosis, and monitoring therapeutic response across degenerative retinal disorders such as AMD and DR. Its ability to detect subtle microcirculatory changes supports its growing role in ophthalmic diagnostics, with a potential key role in personalized treatment planning.

## Data Availability

All data and material are available from the corresponding author.

## Supplementary Materials

The following supporting information can be downloaded at: https://www.mdpi.com/article/10.3390/jcm14248928/s1 [38].

## References

[1] Romano F., Lamanna F., Gabrielle P.H., Teo K.Y.C., Battaglia Parodi M., Iacono P., Fraser-Bell S., Cornish E.E., Nassisi M., Viola F. (2023). Update on retinal vein occlusion. Asia-Pac. J. Ophthalmol..

[2] Jager R.D., Mieler W.F., Miller J.W. (2008). Age-related macular degeneration. N. Engl. J. Med..

[3] Cheung N., Mitchell P., Wong T.Y. (2010). Diabetic retinopathy. Lancet.

[4] Kornblau I.S., El-Annan J.F. (2019). Adverse reactions to fluorescein angiography: A comprehensive review of the literature. Surv. Ophthalmol..

[5] Sugiyama T. (2014). Basic technology and clinical applications of the updated model of laser speckle flowgraphy to ocular diseases. Photonics.

[6] Kunikata H., Nakazawa T. (2016). Recent clinical applications of laser speckle flowgraphy in eyes with retinal disease. Asia-Pac. J. Ophthalmol..

[7] Sugiyama T., Araie M., Riva C.E., Schmetterer L., Orgul S. (2010). Use of laser speckle flowgraphy in ocular blood flow research. Acta Ophthalmol..

[8] Luft N., Wozniak P.A., Aschinger G.C., Fondi K., Bata A.M., Werkmeister R.M., Schmidl D., Witkowska K.J., Bolz M., Garhöfer G. (2016). Ocular blood flow measurements in healthy white subjects using laser speckle flowgraphy. PLoS ONE.

[9] Iwase T., Yamamoto K., Ra E., Murotani K., Matsui S., Terasaki H. (2015). Diurnal variations in blood flow at the optic nerve head and choroid in healthy eyes. Medicine.

[10] Tseng Y.H., Wu P.L., Kang E.Y., Chen K.J., Yeh P.H., Yeung L., Sun M.H., Wang N.K., Hwang Y.S., Lai C.C. (2025). Advancements in ocular blood flow imaging: Clinical applications of laser speckle flowgraphy and optical coherence tomography angiography in retinal and choroidal vascular diseases. Surv. Ophthalmol..

[11] Calzetti G., Fondi K., Bata A.M., Luft N., Wozniak P.A., Witkowska K.J., Bolz M., Popa-Cherecheanu A., Werkmeister R.M., Schmidl D. (2018). Assessment of choroidal blood flow using laser speckle flowgraphy. Br. J. Ophthalmol..

[12] Mursch-Edlmayr A.S., Luft N., Podkowinski D., Ring M., Schmetterer L., Bolz M. (2019). Short-term effect on the ocular circulation induced by unilateral intravitreal injection of aflibercept in age-related maculopathy. Acta Ophthalmol..

[13] Mursch-Edlmayr A.S., Luft N., Podkowinski D., Ring M., Schmetterer L., Bolz M. (2020). Effects of three intravitreal injections of aflibercept on the ocular circulation in eyes with age-related maculopathy. Br. J. Ophthalmol..

[14] Calzetti G., Mora P., Favilla S., Ottonelli G., Devincenzi G., Carta A., Tedesco S., Mursch-Edlmayr A., Garhöfer G., Gandolfi S. (2020). Assessment of choroidal neovascularization perfusion: A pilot study with laser speckle flowgraphy. Transl. Vis. Sci. Technol..

[15] Calzetti G., Mora P., Borrelli E., Sacconi R., Ricciotti G., Carta A., Gandolfi S., Querques G. (2021). Short-term changes in retinal and choroidal relative flow volume after anti-VEGF treatment for neovascular age-related macular degeneration. Sci. Rep..

[16] Kato N., Haruta M., Furushima K., Arai R., Matsuo Y., Yoshida S. (2023). Decrease in ocular blood flow thirty minutes after intravitreal injections of brolucizumab and aflibercept for neovascular age-related macular degeneration. Clin. Ophthalmol..

[17] Takizawa H., Yasuda M., Hoshi K., Okabe T., Kunikata H., Nakazawa T. (2024). Changes in ocular blood flow in patients with neovascular age-related macular degeneration after intravitreal injection of ranibizumab biosimilar and brolucizumab. Int. Ophthalmol..

[18] Linton E.F., Ahmad N.U., Filister R., Wang J.K., Sohn E.H., Kardon R.H. (2025). Laser speckle flowgraphy reveals widespread reductions in ocular blood flow in nonexudative age-related macular degeneration. Am. J. Ophthalmol..

[19] Kato N., Haruta M., Matsuo Y., Dake S., Kojima Y., Arai R., Sato K., Furushima K., Yoshida S. (2025). Comparison of ocular blood flow changes at 30 minutes after intravitreal injection of faricimab, brolucizumab, and aflibercept 2 mg for neovascular age-related macular degeneration. Retina.

[20] Nitta F., Kunikata H., Aizawa N., Omodaka K., Shiga Y., Yasuda M., Nakazawa T. (2014). The effect of intravitreal bevacizumab on ocular blood flow in diabetic retinopathy and branch retinal vein occlusion as measured by laser speckle flowgraphy. Clin. Ophthalmol..

[21] Okamoto M., Matsuura T., Ogata N. (2015). Effects of panretinal photocoagulation on choroidal thickness and choroidal blood flow in patients with severe nonproliferative diabetic retinopathy. Retina.

[22] Shiba C., Shiba T., Takahashi M., Matsumoto T., Hori Y. (2016). Relationship between glycosylated hemoglobin A1c and ocular circulation by laser speckle flowgraphy in patients with/without diabetes mellitus. Graefes Arch. Clin. Exp. Ophthalmol..

[23] Iwase T., Kobayashi M., Yamamoto K., Ra E., Terasaki H. (2017). Effects of photocoagulation on ocular blood flow in patients with severe non-proliferative diabetic retinopathy. PLoS ONE.

[24] Sugimoto M., Nunome T., Sakamoto R., Kobayashi M., Kondo M. (2017). Effect of intravitreal ranibizumab on the ocular circulation of the untreated fellow eye. Graefes Arch. Clin. Exp. Ophthalmol..

[25] Yamada Y., Suzuma K., Onizuka N., Uematsu M., Mohamed Y.H., Kitaoka T. (2017). Evaluation of retinal blood flow before and after panretinal photocoagulation using pattern scan laser for diabetic retinopathy. Curr. Eye Res..

[26] Okamoto M., Yamashita M., Ogata N. (2018). Effects of intravitreal injection of ranibizumab on choroidal structure and blood flow in eyes with diabetic macular edema. Graefes Arch. Clin. Exp. Ophthalmol..

[27] Mikoshiba Y., Iwase T., Ueno Y., Yamamoto K., Ra E., Terasaki H. (2018). A randomized clinical trial evaluating choroidal blood flow and morphology after conventional and pattern scan laser panretinal photocoagulation. Sci. Rep..

[28] Iwase T., Mikoshiba Y., Ra E., Yamamoto K., Ueno Y., Terasaki H. (2019). Evaluation of blood flow on optic nerve head after pattern scan and conventional laser panretinal photocoagulation. Medicine.

[29] Toto L., Evangelista F., Viggiano P., Erroi E., D’Onofrio G., Libertini D., Porreca A., D’Aloisio R., Mariacristina P., Di Antonio L. (2020). Changes in ocular blood flow after ranibizumab intravitreal injection for diabetic macular edema measured using laser speckle flowgraphy. Biomed Res. Int..

[30] Mizui T., Noma H., Yasuda K., Kanemaki T., Goto H., Shimura M. (2020). Intravitreal ranibizumab reduced ocular blood flow and aqueous cytokine levels and improved retinal morphology in patients with diabetic macular edema. Sci. Rep..

[31] Ueno Y., Iwase T., Goto K., Tomita R., Ra E., Yamamoto K., Terasaki H. (2021). Association of changes of retinal vessels diameter with ocular blood flow in eyes with diabetic retinopathy. Sci. Rep..

[32] Saima Y., Yokota H., Kushiyama A., Hanaguri J., Ohno A., Takase K., Sugiyama R., Muranaka K., Yamagami S., Nagaoka T. (2024). Effects of switching from intravitreal injection of aflibercept to faricimab on ocular blood flow in patients with diabetic macular edema. Sci. Rep..

[33] Mizukami T., Mizumoto S., Ishibashi T., Ueno S., Toyonishi T., Tachibana K., Mishima S., Shimomura Y. (2024). Changes in ocular blood flow after intravitreal injection for diabetic macular edema between aflibercept and faricimab. Clin. Ophthalmol..

[34] Abu El-Asrar A.M., Alsarhani W.K., AlBloushi A.F., Alzubaidi A., Gikandi P., Stefánsson E. (2025). Effect of panretinal photocoagulation on retinal oxygen metabolism and ocular blood flow in diabetic retinopathy. Acta Ophthalmol..

[35] Fukumura D., Gohongi T., Kadambi A., Izumi Y., Ang J., Yun C.O., Buerk D.G., Huang P.L., Jain R.K. (2001). Predominant role of endothelial nitric oxide synthase in vascular endothelial growth factor-induced angiogenesis and vascular permeability. Proc. Natl. Acad. Sci. USA.

[36] Hu Y., Wu Q., Liu B., Cao D., Dong X., Zhang L., Li T., Yang X., Yu H. (2019). Comparison of clinical outcomes of different components of diabetic macular edema on optical coherence tomography. Graefes Arch. Clin. Exp. Ophthalmol..

[37] Midena E., Lupidi M., Toto L., Covello G., Veritti D., Pilotto E., Cicinelli M.V., Lattanzio R., Figus M., Midena G. (2025). AI-Assisted OCT Clinical Phenotypes of Diabetic Macular Edema: A Large Cohort Clustering Study. J. Clin. Med..

[38] Page M.J., McKenzie J.E., Bossuyt P.M., Boutron I., Hoffmann T.C., Mulrow C.D., Shamseer L., Tetzlaff J.M., Akl E.A., Brennan S.E. (2021). The PRISMA 2020 statement: An updated guideline for reporting systematic reviews. BMJ.

